# Resveratrol Protects against Zearalenone-Induced Mitochondrial Defects during Porcine Oocyte Maturation via PINK1/Parkin-Mediated Mitophagy

**DOI:** 10.3390/toxins14090641

**Published:** 2022-09-16

**Authors:** Jiehuan Xu, Lingwei Sun, Mengqian He, Shushan Zhang, Jun Gao, Caifeng Wu, Defu Zhang, Jianjun Dai

**Affiliations:** 1 Institute of Animal Husbandry and Veterinary Science, Shanghai Academy of Agricultural Sciences, Shanghai 201106, China; 2 Shanghai Municipal Key Laboratory of Agri-Genetics and Breeding, Shanghai 201106, China; 3 Shanghai Engineering Research Center of Breeding Pig, Shanghai 201106, China; 4 Key Laboratory of Livestock and Poultry Resources (Pig) Evaluation and Utilization, Ministry of Agriculture and Rural Affairs, Shanghai 201106, China

**Keywords:** zearalenone, mitophagy, resveratrol, PINK1/Parkin, porcine oocyte

## Abstract

Mitochondria hold redox homeostasis and energy metabolism as a crucial factor during oocyte maturation, while the exposure of estrogenic mycotoxin zearalenone causes developmental incapacity in porcine oocyte. This study aimed to reveal a potential resistance of phytoalexin resveratrol against zearalenone during porcine oocyte maturation and whether its mechanism was related with PTEN-induced kinase 1 (PINK1)/Parkin-mediated mitophagy. Porcine oocytes were exposed to 20 μM zearalenone with or without 2 μM resveratrol during in vitro maturation. As for the results, zearalenone impaired ultrastructure of mitochondria, causing mitochondrial depolarization, oxidative stress, apoptosis and embryonic developmental incapacity, in which mitophagy was induced in response to mitochondrial dysfunction. Phytoalexin resveratrol enhanced mitophagy through PINK1/Parkin in zearalenone-exposed oocytes, manifesting as enhanced mitophagy flux, upregulated PINK1, Parkin, microtubule-associated protein light-chain 3 beta-II (LC3B-II) and downregulated substrates mitofusin 2 (MFN2), voltage-dependent anion channels 1 (VDAC1) and p62 expressions. Resveratrol redressed zearalenone-induced mitochondrial depolarization, oxidative stress and apoptosis, and accelerated mitochondrial DNA copy during maturation, which improved embryonic development. This study offered an antitoxin solution during porcine oocyte maturation and revealed the involvement of PINK1/Parkin-mediated mitophagy, in which resveratrol mitigated zearalenone-induced embryonic developmental incapacity.

## 1. Introduction

Grains are major ingredients in feed formulation in intensive livestock farms, while around 70% of cereal feeds is polluted by mycotoxins [1]. Zearalenone is a non-steroidal estrogenic mycotoxin produced by multiple species of the *Fusarium* genus that is frequently detected in cereals [2]. Based on a ten-year global survey from more than 74,000 samples in 100 countries, zearalenone is one of the top-three mycotoxins in animal feedstuffs [3]. Serious studies have reported that zearalenone and its metabolites have estrogen-like activities in human and domestic animals that compete with the estrogen receptor [4]. As a result, excessive estrogen syndrome and a series of pathological changes in the reproductive organs occur in domestic animals through ingestion of zearalenone-polluted fodder [5], causing economic loss to husbandry and the culling of breeding stocks.

As a main livestock and medical model, pigs have been considered more sensitive to zearalenone toxicity [6]. High-dose zearalenone intake causes permanent pathologic alterations in pigs, leading to edema and prolapse of the vagina, infertility, pseudopregnancy and embryo lethal resorption [7]. The toxicity of zearalenone on porcine oocytes is presumed to relate to oxidative stress, apoptosis, autophagy and epigenetic modification variation [8]. Similar research on porcine blastocysts also claimed that zearalenone exposure promoted DNA damage, apoptosis and autophagy [9]. Other studies demonstrated that zearalenone exposure impaired mitochondrial membrane potential (ΔΨm) [10], causing oxidative stress through the mitochondrial apoptosis pathway in pigs [11]. These studies hinted the state of mitochondria as a presumable toxicology of zearalenone, while in this study, further disclosure of their interrelation and potential rescue solution of in vitro maturation (IVM) oocytes will be evaluated.

Each oocyte equips a large supply of mitochondria to meet the energy requirement of maturation and embryonic development. Mitochondria are a crucial factor determining the developmental competence of oocytes, as they control intracellular Ca^2+^ homeostasis and produce ATP for continuous transcription and translation [12]. During mitochondrial renewal or mitochondrial stress, mitophagy, oocytes selectively degrading impaired or supernumerary mitochondria through autophagy, are involved in maintaining mitochondrial homeostasis and oocyte survival [13].

As an evolutionarily conserved intracellular function, autophagy is considered a stress-responsive autonomous process. Cells selectively dispose of redundant, potentially harmful or dysfunctional cytoplasmic entities via autophagy to maintain intracellular homeostasis [14]. Mitophagy, selectively eliminating mitochondria via autophagy, is critical for the quality and quantity control of mitochondria. Impaired mitochondria are targeted by the autophagic system to form mitophagosomes and subsequently delivered to lysosomes for degradation. A primary signaling pathway to regulate mitophagy is through ubiquitin-dependent PTEN-induced kinase 1 (PINK1)/Parkin. PINK1, a Ser/Thr kinase, stabilizes on depolarized mitochondria and phosphorylates mitofusin (MFN), voltage-dependent anion channels 1 (VDAC1) and other substrates on the outer-mitochondrial membrane (OMM) [15,16]. E3 ubiquitin ligase Parkin is then recruited by phosphorylated substrates from cytoplasm to depolarized mitochondria and catalyzes the transferring of ubiquitin to substrates on OMM [17,18]. These polyubiquitinated substrates are recognized by p62 and interact with microtubule-associated protein light chain 3 (LC3) through LC3-interacting region motif and further cause the bilayer membrane structure to encapsulate damaged mitochondria. The mitophagosome is then formed. In order to degrade target mitochondria as its cargo inside, fashioned mitophagosome subsequently fused with lysosome, forming mitophagolysosome. Target mitochondria are degraded into matrix substances in the acidic environment of lysosomes and recycled by cell.

Due to cytoplasmic immaturity, the developmental capacity of IVM oocytes is considered lower than ovulatory oocytes [19]. Therefore, numerous antioxidants have been evaluated to rescue IVM oocytes. Resveratrol, a natural phytoalexin, has been widely studied for its resistant properties against oxidation, carcinogenicity and inflammation. A previous study demonstrated multiple effects of resveratrol towards oocytes, such as enhancing the clearance of mitochondrial damage [20], protecting oocytes form oxidative stress and apoptosis, promoting oocyte maturation and hindering postovulatory aging [21]. Recent studies revealed that these beneficial effects are closely related to autophagy/mitophagy indued by resveratrol through multiple signaling pathways. For example, mitophagy and enhanced mitochondrial protein FOXO3a induced by resveratrol was considered as a potential mechanism against postovulatory oocyte aging [22]. In consideration of the antitoxin effect of resveratrol and its close relation with mitophagy, this study presumed a hypothesis that resveratrol could rescue zearalenone-induced maturation impairment of porcine oocyte through PINK1/Parkin-mediated mitophagy. Resveratrol was concurrently co-incubated with zearalenone during porcine oocyte maturation for the hypothetical resistance against zearalenone.

The purpose of this study was to (1) elucidate whether resveratrol could alleviate zearalenone-indued oxidative stress, apoptosis and developmental incapacity during porcine oocyte maturation through mitophagy and (2) clarify whether the PINK1/Parkin signaling pathway was involved in it.

## 2. Results

### 2.1. Resveratrol Alleviated Zearalenone-Induced Embryonic Developmental Failures

**Figure 1:**
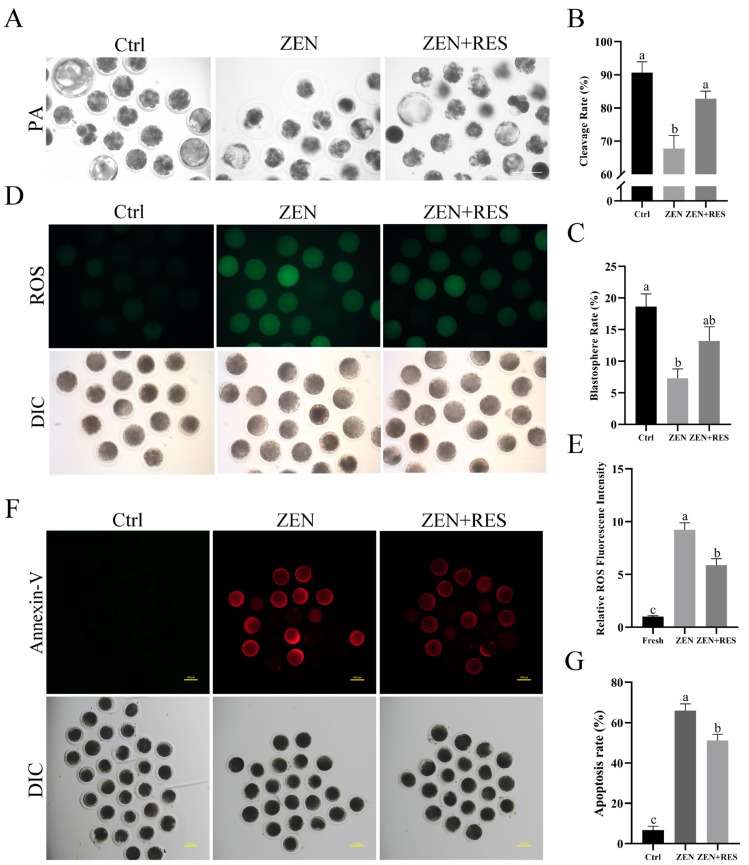
Resveratrol alleviated zearalenone-induced oocyte oxidative stress, early apoptosis and loss of embryonic developmental potential. (**A**) Morphological observation of PA embryos in 7 d. Bar = 100 μm. (**B**,**C**) Statistical analysis on the ratio of cleavage and blastosphere number. (**D**) Determination of ROS generation. Bar = 100 μm. (**E**) Statistical analysis of ROS generation. (**F**) Fluorescence observation of oocyte apoptosis. Bar = 100 μm. (**G**) Statistical analysis of apoptosis rates. Typical images of each different treatment towards oocytes were present. Independent replications were performed three times in each experiment. Different lowercase letters on the statistical graphs indicated significant differences between treatments (*p*-value < 0.05).

To clarify the effect of estrogen-like mycotoxin zearalenone and the hypothetical antitoxin effects of resveratrol against zearalenone during porcine oocyte maturation, parthenogenetic activation (PA) and in vitro zygote culture were performed to determine the cleavage and blastosphere rates as embryonic developmental potential (Figure 1A–C). The results revealed that zearalenone hindered the development of PA embryo, demonstrated as significantly decreased cleavage rate (90.67% ± 3.24% vs. 67.74% ± 3.96%, *p* < 0.05) and blastosphere rate (18.64% ± 1.98% vs. 7.28% ± 1.50%, *p* < 0.05). Compared to zearalenone-exposed oocytes, the cleavage rate (67.74% ± 3.96% vs. 82.78% ± 2.31%, *p* < 0.05) and blastosphere rate (7.28% ± 1.50% vs. 13.20% ± 2.23%) raised up when resveratrol co-incubated with zearalenone during oocyte maturation.

### 2.2. Resveratrol Alleviated Zearalenone-Induced Oxidative Stress and Apoptosis during Oocyte Maturation

To reveal the mechanism of embryonic developmental failures induced by zearalenone and the antitoxin effect of resveratrol, oocyte ROS levels (Figure 1D,E) and apoptosis rates (Figure 1F,G) were, respectively, determined. Compared to maturated oocytes, zearalenone significantly upregulated the ROS generation (1.00 ± 0.08 vs. 9.23 ± 0.67, *p* < 0.05) and apoptosis rate (6.67% ±1.93% vs. 66.02% ± 3.31%, *p* < 0.05) in porcine oocytes, while, compared to zearalenone-exposed oocytes, resveratrol significantly inhibited the ROS generation (9.23 ± 0.67 vs. 5.89 ± 0.60, *p* < 0.05) and apoptosis (66.02% ± 3.31% vs. 51.25% ± 3.08%, *p* < 0.05) when it co-incubated with zearalenone during oocyte maturation.

### 2.3. Resveratrol Alleviated Zearalenone-Induced Mitochondrial Defects during Oocyte Maturation

**Figure 2:**
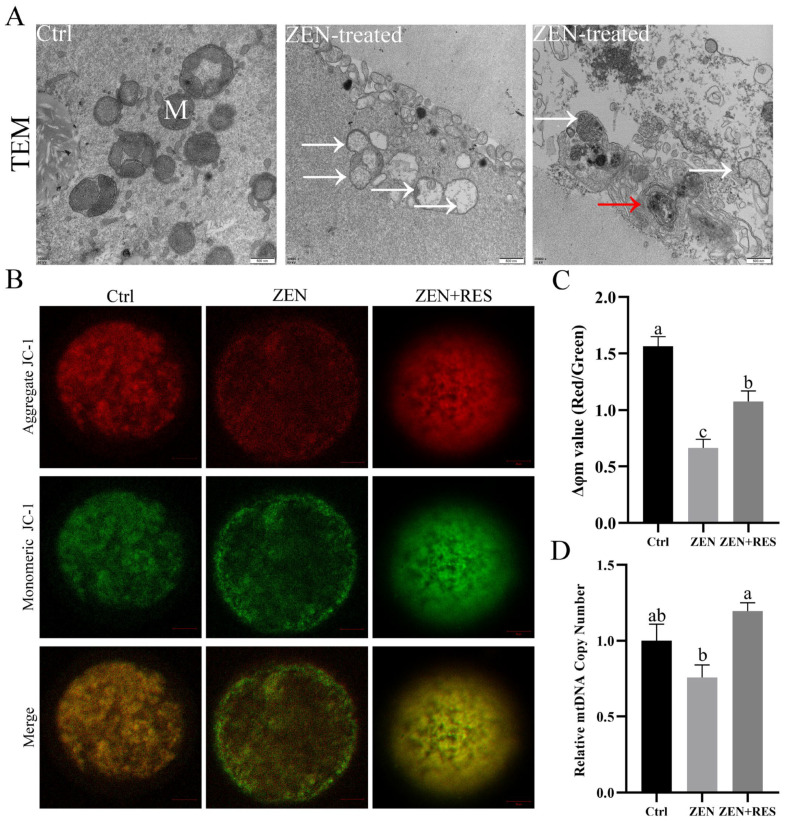
Resveratrol alleviated zearalenone-induced mitochondrial dysfunction during porcine oocyte maturation. (**A**). Mitochondrial ultrastructure of zearalenone-exposed porcine oocytes was observed by transmission electron microscopy. M: mitochondria. White arrows: damaged mitochondria with vague or disabled cristae. Red arrow: mitophagosome. Bar = 500 nm. (**B**) Detection of ΔΨm. Red fluorescence represented high ΔΨm while green fluorescence represented low ΔΨm. Bar = 20 μm. (**C**) Statistical analysis of ΔΨm value (Red/Green). (**D**) Comparison on relative mtDNA copy numbers. Relative gene expression of NADH dehydrogenase subunit 1 (ND1) was determined by real-time PCR. Typical ultrastructural images or intracellular fluorescence images were given representing different oocyte treatments. Independent replications were performed three times in each treatment. Different lowercase letters on the statistical graphs indicated significant differences between treatments (*p*-value < 0.05).

To explore the effect of zearalenone towards oocyte mitochondrial state, ultrastructure observation and determinations of ΔΨm and mitochondrial DNA (mtDNA) copy numbers were performed. As for the results, numerous mitochondria in regular morphology with mitochondrial crista were equably distributed in the cytoplasm in maturated porcine oocytes. After zearalenone exposure, the destruction of mitochondrial internal structure, including vacuolated mitochondria and vague or disabled cristae (white arrows), was observed in oocytes. Typical structure of mitophagosome (red arrow) was observed in zearalenone-exposed oocytes (Figure 2A), which indicated the activation of mitophagy.

In the meantime, the ultrastructural damage caused by zearalenone led to the variation in ΔΨm (Figure 2B,C) and relative mtDNA copy number (Figure 2D), which were determined by fluorescence probe JC-1 and realtime-PCR. As for the results, compared to maturated oocytes, the ΔΨm (1.56 ± 0.08 vs. 0.66 ± 0.08, *p* < 0.05) and relative mtDNA copy number (1.00 ± 0.11 vs. 0.76 ± 0.08) were both decreased in zearalenone-exposed oocytes. The results above revealed that zearalenone exposure led to mitochondrial structural damage, depolarization and decreased mtDNA copy number and also hinted the involvement of mitophagy in zearalenone-induced mitochondrial defects during porcine oocyte maturation.

To explore the effect of resveratrol on mitochondrial defects against zearalenone, ΔΨm and relative mtDNA copy numbers were determined when resveratrol was co-incubating with zearalenone during porcine oocyte maturation (Figure 2B–D). As for the results, resveratrol significantly upregulated the ΔΨm (0.66 ± 0.08 vs. 1.08 ± 0.09, *p* < 0.05) and relative mtDNA copy number (0.76 ± 0.08 vs. 1.20 ± 0.05, *p* < 0.05) in zearalenone-exposed oocytes. The results reveled that resveratrol alleviated zearalenone-induced mitochondrial defects in porcine oocytes during maturation.

### 2.4. Resveratrol Enhanced Mitophagy Flux during the Maturation of Zearalenone-Exposed Oocytes

**Figure 3:**
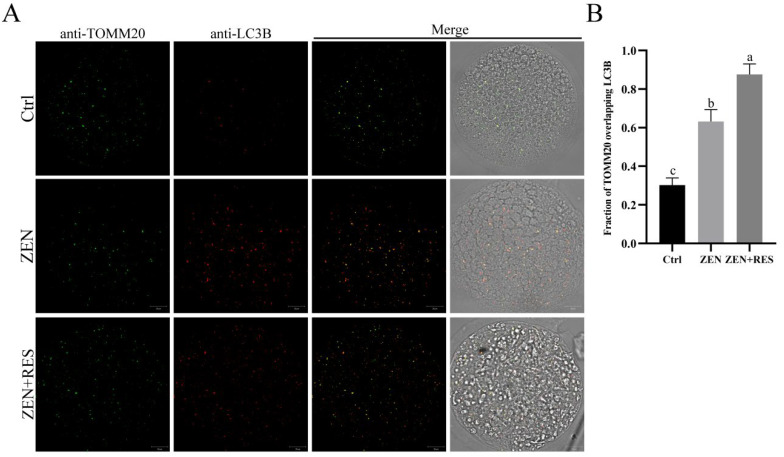
Fluorescence colocalization of mitophagosomes in porcine oocytes. (**A**) Fluorescence colocalization between VDAC1 and LC3B. Bar = 20 μm. (**B**) Statistical analysis of the fraction of VDAC1 overlapping LC3B. Typical images of each different treatment towards oocytes were present. Independent replications were performed three times. Different lowercase letters on the statistical graphs indicated significant differences between treatments (*p*-value < 0.05).

To reveal the mechanism of resveratrol alleviating zearalenone-induced mitochondrial dysfunction and the effect of resveratrol on mitophagy flux in zearalenone-exposed oocytes, the formation and degradation of mitophagosomes were, respectively, determined. Representing the formation of mitophagosomes, mitochondrial outer-membrane marker translocase of outer-mitochondrial membrane 20 (TOMM20) and autophagic vacuole marker microtubule-associated protein 1 light-chain 3 beta (LC3B) were stained using immunofluorescence and colocalized under a laser scanning confocal microscope (LSCM) (Carl Zeiss, Jena, Germany) (Figure 3). After oocyte exposing to zearalenone during maturation, the fraction of TOMM20 overlapping LC3B significantly raised up (0.30 ± 0.04 vs. 0.63 ± 0.06, *p* < 0.05). Compared to it, resveratrol further upregulated the formation of mitophagosomes in zearalenone-exposed oocytes, demonstrating a significantly enhanced fraction of TOMM20 overlapping LC3B (0.63 ± 0.06 vs. 0.88 ± 0.05, *p* < 0.05).

**Figure 4:**
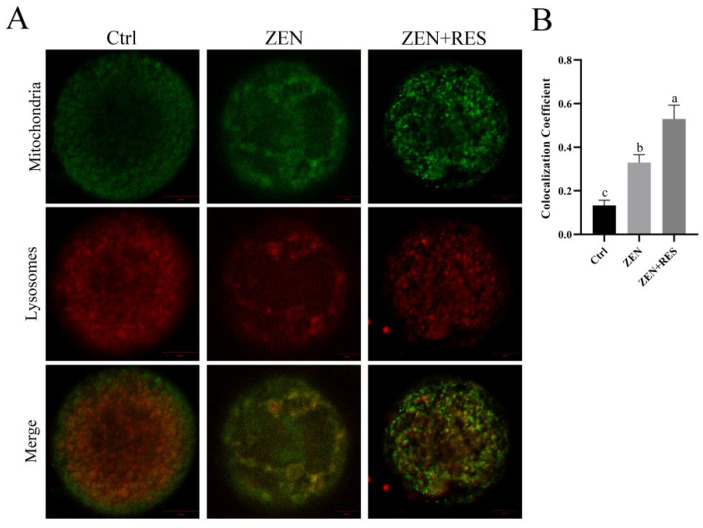
Fluorescence colocalization of mitophagolysosomes in porcine oocytes. (**A**) The fluorescence of mitochondria (represented in green) and lysosomes (represented in red) was inspected by LSCM. Bar = 20 μm. Typical images of each different treatment towards oocytes were present. Independent replications were performed three times. (**B**) Statistical analysis of colocalization coefficient. Different lowercase letters on the statistical graphs indicated significant differences between treatments (*p*-value < 0.05).

Representing the formation of mitophagolysosomes, the fluorescence colocalization of mitochondria and lysosomes was stained by fluorescence probes Mito-tracker green and Lyso-tracker red and observed under LSCM (Figure 4). The fluorescence dots of mitochondria and lysosomes equally distributed in maturated oocytes, showing a loose bond between mitochondria and lysosomes. Compared to it, the colocalization coefficient between mitochondria and lysosomes significantly upregulated in zearalenone-exposed oocytes (0.13 ± 0.02 vs. 0.33 ± 0.04, *p* < 0.05), which revealed the enhanced formation of mitophagolysosomes induced by zearalenone. Compared to zearalenone-exposed oocytes, the colocalization coefficient was further upregulated (0.33 ± 0.04 vs. 0.54 ± 0.06, *p* < 0.05) when resveratrol co-incubated with zearalenone during oocyte maturation.

The results above revealed that mitophagy was activated in zearalenone-exposed porcine oocytes. Based on it, antitoxin resveratrol further enhanced mitophagy flux in zearalenone-exposed oocytes.

### 2.5. Resveratrol Enhanced Mitophagy through PINK1/Parkin Signaling Pathway in Zearalenone-Exposed Oocytes

**Figure 5:**
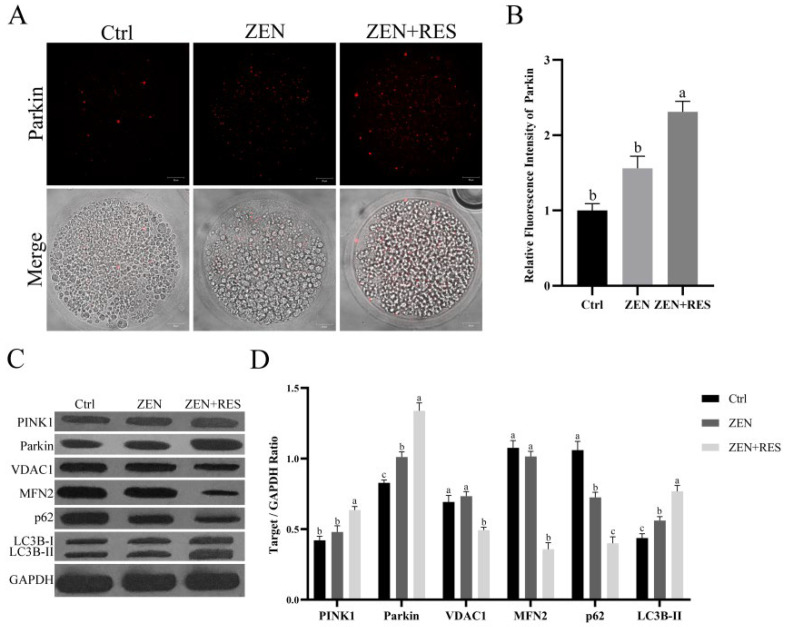
Resveratrol enhanced PINK1/Parkin signaling pathway in zearalenone-exposed porcine oocytes. (**A**) Fluorescence localization of Parkin. Bar = 20 μm. Typical intracellular fluorescent distributions were given representing different oocyte treatments. (**B**) Statistical analysis of relative fluorescence intensity of Parkin. (**C**) Protein expressions of PINK1/Parkin-mediated mitophagy determined by Western blotting. (**D**) Statistical analysis of protein expressions. Independent replications were performed three times in each experiment. Different lowercase letters on the statistical graphs indicated significant differences between treatments (*p*-value < 0.05).

To reveal the mechanism of resveratrol altering mitophagy flux in zearalenone-exposed oocytes, the fluorescence aggregation of Parkin and the protein expressions of PINK1/Parkin, a primary signaling pathway of mitophagy, were determined by immunofluorescence and Western blotting. The results showed that, compared to zearalenone-exposed oocytes, resveratrol significantly enhanced fluorescence aggregation of Parkin during oocyte maturation (1.56 ± 0.16 vs. 2.31 ± 0.14, *p* < 0.05) (Figure 5A,B).

Meanwhile, the results of the Western blot assay revealed that mitophagy was induced by zearalenone in porcine oocyte during maturation (Figure 5B,C), demonstrated as significantly upregulated autophagy marker LC3B-II (0.44 ± 0.03 vs. 0.56 ± 0.03, *p* < 0.05), and p62 degradation (1.06± 0.06 vs. 0.72 ± 0.04, *p* < 0.05), comparing zearalenone-exposed oocytes to maturated oocytes. Meanwhile, compared to zearalenone-exposed oocytes, resveratrol upregulated the protein expressions of the PINK1/Parkin signaling pathway, demonstrated as significantly upregulated PINK1 (0.48 ± 0.04 vs. 0.64 ± 0.03, *p* < 0.05), Parkin (1.01 ± 0.04 vs. 1.34 ± 0.06, *p* < 0.05), significant degradation of ubiquitinated substrates VDAC1 (0.73 ± 0.03 vs. 0.49 ± 0.02, *p* < 0.05) and MFN2 (1.02 ± 0.04 vs. 0.36 ± 0.05, *p* < 0.05) and significantly upregulated downstream autophagy marker LC3B-II (0.56 ± 0.03 vs. 0.77 ± 0.04, *p* < 0.05) and p62 degradation (0.73 ± 0.04 vs. 0.40 ± 0.05, *p* < 0.05).

## 3. Discussion

As a non-steroidal estrogenic mycotoxin, zearalenone is ingested by domestic animals through grains and cereal products and generally causes hypofertility and considerable financial loss in animal husbandry. A previous study on porcine oocytes attributed the toxicity of zearalenone as impairing the function and distribution of organelles, interdicting the extrusion of polar body and disturbing the expansion of cumulus granulosa cells [10]. In vivo research showed that 1.04 mg/kg zearalenone environmental intake induced follicular atresia in pigs, inhibiting proliferation of porcine granulosa cells and inducing its apoptosis and necrosis [23]. However, the dose of zearalenone enriched to porcine follicle fluid was still required to be clarified. On the other side, in vitro dose test on porcine cumulus–oocyte complexes revealed that 10 μM to 30 μM zearalenone incubation led to a significant decline in oocyte maturation rate [8] and it further decreased to less than 5% when the concentration of zearalenone up to 50 μM [24]. A similar study on mouse oocytes also claimed that zearalenone disturbed G2/M transition [25]. In spite of this evidence, however, oxidative-stress-mediated apoptosis was also considered as a plausible mechanism of zearalenone toxicity [9,26], which hinted a potential toxicity effect towards mitochondria and the application of antioxidants. In consideration of these studies, we cultured cumulus–oocyte complexes supplied with 20 μM zearalenone to explore its effect on the mitochondrial function of porcine oocytes and the potential rescue mechanism. Therefore, in this study, we first investigated the outcome from oocyte meiosis to PA embryo, verifying the fate of oocytes that zearalenone exposure during IVM led to the failure of cleavage and blastosphere formation, whereas the application of resveratrol gained higher embryonic developmental capacity of PA oocytes (Figure 1A–C), which led to further investigations of the antitoxin effects and their mechanism.

In this study, zearalenone was confirmed to disrupt mitochondrial homeostasis, demonstrating structural destruction, depolarization and DNA damage in mitochondria (Figure 2). Dysfunctional mitochondria are less competent to counteract ROS production and the supraphysiological levels of ROS in zearalenone-exposed oocyte led to oxidative stress (Figure 1D,E). Mitochondria are not only the primary endogenous source of ROS synthesis and release, but also the main target organelle of ROS effects. ROS increases the permeability of the inner-mitochondrial membrane to solutes through activating the mitochondrial permeability transition pore [27], which induces mitochondrial depolarization. Therefore, in this study, zearalenone-induced supraphysiological ROS and structural damage of mitochondria caused a vicious loop between oxidative stress and aggravated mitochondrial dysfunction, in which apoptosis was activated. In addition, zearalenone was previously proved to induce apoptosis and/or autophagy in oocytes [10], spermatogonia [28] and embryo [29], whereas, in this study, we provided evidence that mitophagy was activated in response to zearalenone-induced mitochondrial defects and oxidative stress in porcine oocyte, demonstrated as enhanced mitophagy flux (Figure 3 and Figure 4).

Several studies attempted to mitigate zearalenone-induced impairment and oxidative stress in vivo or in vitro. In vivo research claimed that resveratrol was efficacious in reducing DNA lesions and the modulation of antioxidant enzymes caused by zearalenone intake in rats [30]. In vitro comparison in aged mice and humans revealed that about 1.0 μm was a more appropriate concentration of resveratrol than 0.1 μm and 10 μm in IVM medium that induced oocyte maturation and benefitted mitochondrial quality [31]. Meanwhile, another comparison study further revealed that 2 μM was the optimum concentration of resveratrol in a range of 0 to 4 μM to gain increased first polar body extrusion rate and hinder postovulatory aging [21]. A similar study in pigs claimed that reductant Vitamin C prevented hormonal disorders and vulval deformities caused by zearalenone intake [32]. In vitro research claimed that melatonin ameliorated oxidative stress, aberrant mitochondria distribution and DNA damage in zearalenone-exposed porcine embryo [29]. In the meantime, we confirmed that resveratrol relieved mitochondrial dysfunction (Figure 2) and prevented oxidative-stress-induced apoptosis (Figure 1) in zearalenone-exposed oocytes through PINK1/Parkin-mediated mitophagy (Figure 3, Figure 4 and Figure 5).

The progress of apoptosis as well as excessive autophagy generally lead to cell death, while a certain extent of autophagy contributes to maintaining cellular metabolism and environmental homeostasis. In this study, we considered mitophagy as a self-healing mechanism in response of zearalenone infringement towards mitochondria, of which the positive effects of resveratrol-induced autophagy/mitophagy were demonstrated in multiple studies. Resveratrol was verified to enhance SIRT1-mediated autophagy in oocytes against aging [33], alleviating the disorder of mitochondrial biogenesis against benzo(a)pyrene toxicity by promoting mitophagy [34]. A similar effect was also confirmed in flavonoid quercetin, which improved IVM outcomes in porcine by promoting mitophagy, improving mitochondrial function and reducing oxidative stress [35].

Parkin, an E3 ubiquitin ligase belonging to the RING-between-RING family, was involved in the inducement of multiple nerve diseases, including Parkinson’s [36], Alzheimer’s [37] and Huntington disease [38], of which the pathogenesis was closely related with mitochondrial dysregulation. Moreover, it brought about widespread attention whether the deficiency of Parkin-mediated mitophagy might be a potential pathogenesis of these diseases [39]. Nevertheless, the implementation of PINK1/Parkin-mediated mitophagy was ubiquitin dependent. A noteworthy fact was that MFN1 and MFN2, the ubiquitinated substrates of PINK1/Parkin, were recognized as key regulators of mitochondrial fusion in mammals. Although mitochondria were considered morphologically static organelles, they continuously change their shape in response to a variety of cellular signals, which were known as mitochondrial dynamics [40]. Damaged mitochondria may lose their inner-membrane potential, causing accumulation of toxic ROS, then contaminating other mitochondria through mitochondrial fusion. In this study, the enhanced degradation of MFN2 through the ubiquitin–proteasome proteolytic system was determined when resveratrol co-incubated with zearalenone during IVM, which revealed a synchronization between enhanced clearance through mitophagy and inhibited fusion of damaged mitochondria as the antitoxin effect of resveratrol against zearalenone in porcine oocytes.

This in vitro study gave the evidence of phytoalexin resveratrol application showing reproductive toxicity resistance in pigs. However, each oocyte was suspended in follicular fluid of a follicle in ovaries, so the concentration of resveratrol from in vitro to in vivo needs to consider the absorptivity of RES from blood to follicular fluid and its accumulation effect. The bioavailability of oral RES still needs further in vivo studies, since many factors affect its absorption, such as different species, the amount of fat in the diet and drug-delivery methods (oral vs. intraperitoneal injection) et al. [41].

Nevertheless, it occurred with high frequency when livestock underwent a co-exposure between zearalenone and deoxynivalenol. The ecology of deoxynivalenol production often mirrors that of zearalenone, since it is produced by the same fungi and usually detected in crops at the same time. A similar reproductive toxic effect to zearalenone was also detected, of which the exposure of deoxynivalenol towards oocyte hindered the normal progression of meiosis by disrupted meiotic spindle. The inducement of autophagy and apoptosis was also detected in oocytes after deoxynivalenol exposure [42]. Moreover, their co-exposure toward oocytes was confirmed to alter DNA methylation levels [43], which caused the loose embryo developmental capacity [44]. Therefore, whether resveratrol would show a similar resistant effect against deoxynivalenol or their joint toxicity in oocytes through mitophagy remains a worthy research issue.

## 4. Conclusions

We attributed the oxidative stress and apoptosis of porcine oocytes to functional defects in mitochondria induced by zearalenone during IVM, which further led to embryonic developmental incapacity. Phytoalexin resveratrol alleviated zearalenone-induced mitochondrial dysfunction, oxidative stress and apoptosis by upregulating PINK1/Parkin-mediated mitophagy, which improved embryonic developmental potential. This study proposed a feasible protocol for porcine oocytes to resist reproductive toxicity of estrogenic mycotoxin zearalenone with the application of phytoalexin resveratrol during oocyte maturation.

## 5. Materials and Methods

### 5.1. Chemicals

Chemicals applied in this study were purchased from Sigma-Aldrich (St Louis, MO, USA) unless otherwise specified.

### 5.2. Oocyte Maturation

Prepubertal gilts were slaughtered in a local slaughter house. Their ovaries were subsequently separated form enterocoelia, preserved in 0.9% (*w/v*) saline at 39 °C and transported to laboratory within 1 h. A 10 mL syringe (combining with an 18-gauge needle) was used to aspirate porcine follicular fluid (PFF) from antral follicles, which contained cumulus–oocyte complexes (COCs). After being naturally precipitated for 30 min at 39 °C, the superstratum was centrifuged at 3000 r/min for 15 min and filtered to gain PFF, while the sediment was inspected under microscopy to gain COCs. Every 55 COCs were cultured in a well of four-well dishes, with each well consisting of 500 μL in vitro maturation medium [45] and covered with 200 μL mineral oil. 20 μM zearalenone (Pribolab Pte. Ltd., Singapore) with or without 2 μM resveratrol added into maturation medium. The COCs were cultured at 39 °C in a humidified atmosphere of 5% CO_2_ for 44 h and transferred into 0.1% hyaluronidase to stripped oocyte form COCs.

### 5.3. Embryonic Developmental Capacity

PA was performed on cumulus-denuded oocytes after 44 h maturation so as to verify their embryonic developmental capacity. After being washed with PA medium [46] 3 times, cumulus-denuded oocytes were transferred to a microslide 0.5 mm fusion chamber (Model 450, BTX, Holliston, MA, USA) and went through a 1.2 kV/cm direct current pulse in 60 ms by BTX2001 (BTX, Holliston, MA, USA). About 150 electric activated oocytes were cultured in porcine zygote medium-3 [47] at 39 °C in a humidified atmosphere of 5% CO_2_. Number of cleavages was observed and counted at 2 d. Number of blastospheres was counted at 7 d.

### 5.4. Oocyte ROS Level

Next, 30 oocytes were stained by 50 μM 2′,7′-dichlorofluorescin diacetate (DCFH-DA) (S0033, Beyotime, Shanghai, China) for 30 min at 39 °C in a humidified atmosphere of 5% CO_2_, then washed 3 times with PBS and inspected by a fluorescence microscope (Olympus, Tokyo City, Japan). Relative fluorescence intensity was analyzed by image J.

### 5.5. Oocyte Apoptosis

Then, 30 oocytes were stained by 2.5% Annexin V-mCherry (C1069, Beyotime, China) for 30 min at 39 °C in a humidified atmosphere of 5% CO_2_, then washed 3 times with PBS and inspected under fluorescence microscope. Oocytes observed with red fluorescence were considered as apoptotic oocytes.

### 5.6. Intracellular Ultrastructure Observation

The procedures referred to the previous study [46]. In brief, 500 oocytes were fixed in 2.5% glutaraldehyde, embedded in 4% agar, fixed with 1% osmium tetroxide, fully dehydrated with a series of increased concentration of ethanol, replaced with propylene oxide, transferred in Epon-812 and polymerized in polymerization reactor. Ultrathin sections were sliced to form semithin sections, stained with uranyl acetate-lead citrate and observed under transmission electron microscopy.

### 5.7. Mitochondrial Membrane Potential

Following this, 30 oocytes were stained with 5,5′,6,6′-tetrachloro-1,1′,3,3′-tetraethyl-imidacarbocyanine iodide (JC-1) (C2006, Beyotime, China) for 30 min in a humidified atmosphere of 5% CO_2_ to determine the ΔΨm. After staining, oocytes were washed with PBS 3 times, inspected under an LSCM and visualized using software ZEN (Carl Zeiss, Germany). When the ΔΨm was low, JC-1 was kept as monomer which could be detected in green fluorescence, while it formed into aggregation in mitochondria with high ΔΨm and could be detected as red fluorescence. The ratio of red (JC-1 aggregation) to green (JC-1 monomer) fluorescence intensity represented the ΔΨm.

### 5.8. Relative mtDNA Copy Number

Next, 100 oocytes were pooled as one sample for total RNA isolation using TaKaRa MiniBEST Universal RNA Extraction Kit (9767, Takara, Kusatsu, Shiga, Japan). PrimeScript RT Master Mix (RR036A, Takara, Japan) and TB Green^®^ Premix Ex Taq™ II (Tli RNaseH Plus, Takara, Japan) were used for reverse transcription and real-time PCR following manufacturers’ instruction. ND1, specific primer for coding region of mitochondria DNA, was designed on behalf of mitochondrial DNA copy numbers. Glyceraldehyde-3-phosphate dehydrogenase (GAPDH) was designed as reference gene. The primer sequences were listed as follows. ND1, Forward: TCCTACTGGCCGTAGCATTCCT; Reword: TTGAGGATGTGGCTGGTCGTAG. GAPDH, Forward: CGATGGTGAAGGTCGGAGTG; Reword: TGCCGTGGGTGGAATCATAC. The results were calculated by 2^−ΔΔCt^ method.

### 5.9. Immunofluorescence

Oocytes were fixed in 4% paraformaldehyde for 30 min, washed 3 times with 0.1% Tween-20 and transferred to 0.1% Triton X-100 overnight for permeabilization. Permeabilized oocytes were washed 3 times and then blocked with 1% BSA for 1 h.

Blocked oocytes were successively incubated with the primary and secondary antibodies Rabbit anti-Parkin antibody (ab233434, Abcam, Boston, MA, USA) and Goat anti-rabbit IgG H&L (Alexa Fluor^®^ 647) (ab150079, Abcam, USA), washed with PBS 3 times and the fluorescence aggregation of Parkin was observed with LSCM.

For colocalization of LC3B and TOMM20, blocked oocytes were incubated with Rabbit Anti-LC3B (ab192890, Abcam, USA) and Mouse Anti-TOMM20 (ab283317, Abcam, USA) for 1 h, then incubated with secondary antibodies Goat anti-mouse IgG H&L (Alexa Fluor^®^ 488) (ab150113, Abcam, USA) and Goat anti-rabbit IgG H&L (Alexa Fluor^®^ 647) (ab150079, Abcam, USA), washed with PBS 3 times and observed with LSCM. The overlapping coefficient was analyzed using Image J as the fraction of VDAC1 overlapping LC3B.

### 5.10. Fluorescent Colocalization of Mitophagolysosomes

Then, 30 oocytes were co-stained with Mito-Tracker Green (C1048, Beyotime, China) and Lyso-Tracker Red (C1046, Beyotime, China) following the manufacturer’s instructions. Oocytes were then washed 3 times with PBS and observed under LSCM. The colocalization coefficient between the fluorescence of mitochondria and lysosomes was analyzed as the Pearson’s coefficient using Image J software, which represented mitophagolysosomes.

### 5.11. Protein Quantification by Western Blot Assay

For protein extraction, 100 oocytes were gathered and transferred in protein lysis buffer as a sample, with three duplicates for each group and the determination of protein concentration was performed using a BCA Protein Assay Kit (02912E, Cwbiotech, Beijing, China). Western blotting assay was subsequently performed following manufacturers’ protocols to ensure the levels of protein expression. The information of primary and secondary antibodies is listed as follows: Rabbit Anti-PINK1 (ab23707, Abcam, USA), Rabbit anti-Parkin (ab233434, Abcam, USA), Rabbit Anti-VDAC1/Porin (ab15895, Abcam, USA), Rabbit anti-Mitofusin 2 (ab124773, Abcam, USA), Rabbit Anti-LC3B, Rabbit Anti-SQSTM1/p62 (ab233207, Abcam, USA), Rabbit Anti-GAPDH (ab9484, Abcam, USA) and the secondary antibody Goat anti-rabbit IgG (H+L) (HRP) (111-035-003, Jackson, West Grove, PA, USA). The expression of GAPDH was determined as a loading control.

### 5.12. Statistical Analysis

At least three independent replicates were performed in each experiment. Image J was used to determine fluorescence intensity, colocalization coefficient and Western blot quantification. ANOVAs with Duncan multiple comparisons were executed for data comparisons in SPSS Statistics 22 (IBM, Armonk, NY, USA). The results were provided as the mean ± SEM. Different lowercase letters on the statistical graph indicate significant differences between treatments (*p*-value < 0.05).

## Data Availability

Not applicable.
